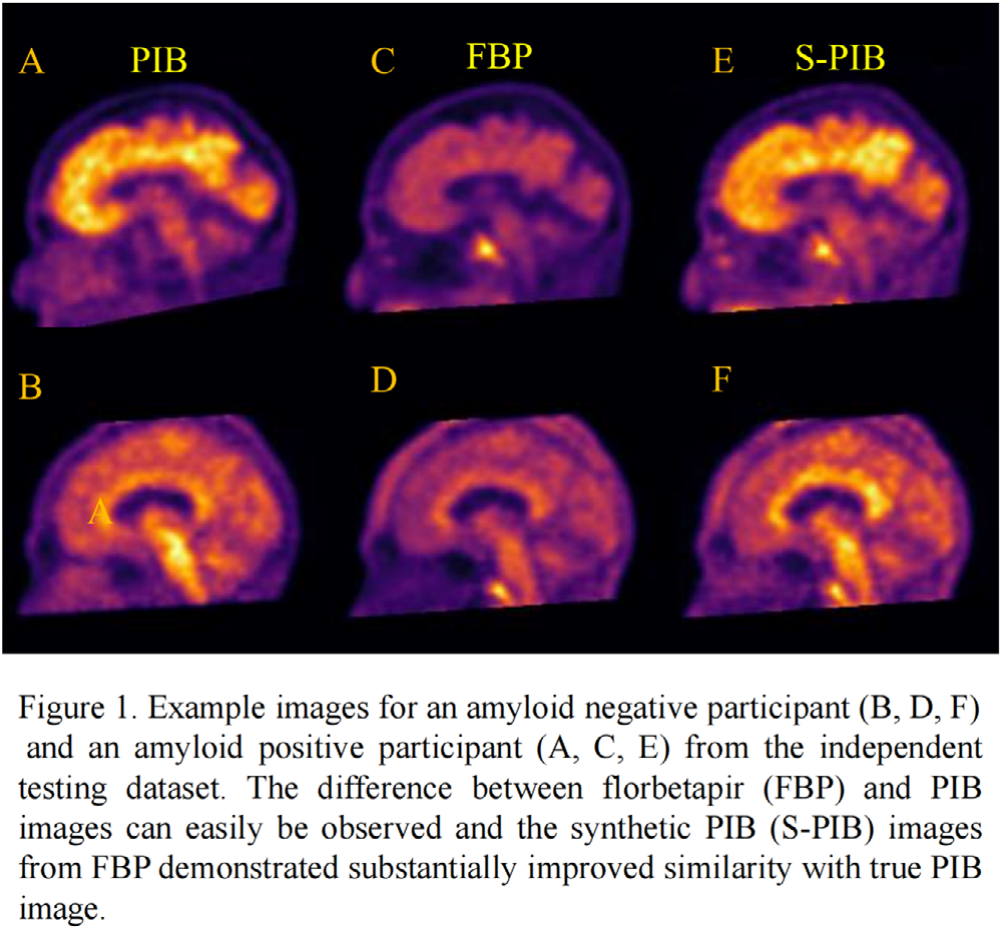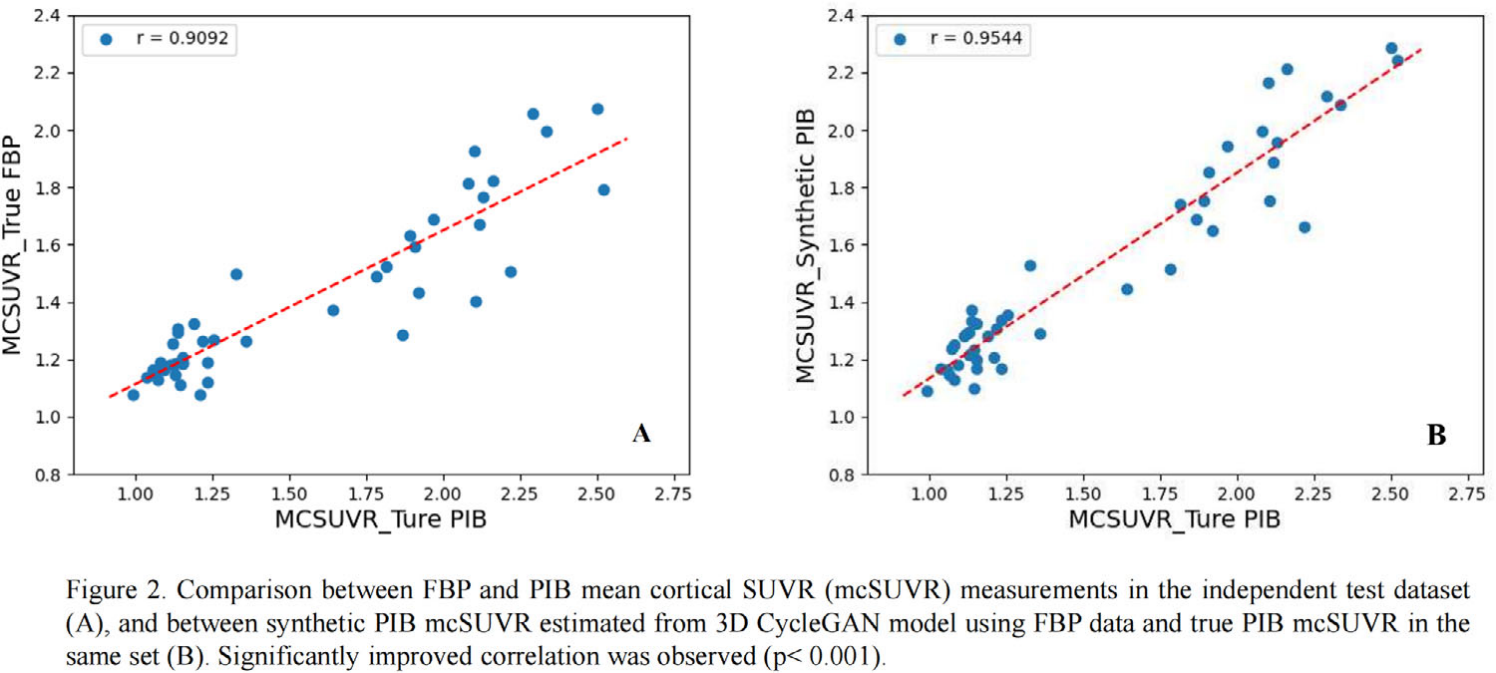# End‐to‐End 3D CycleGAN Model for Amyloid PET Harmonization

**DOI:** 10.1002/alz.095403

**Published:** 2025-01-09

**Authors:** XUANZHAO DONG, Yalin Wang, Jay Shah, Valentina Ghisays, Ji Luo, Yinghua Chen, Wendy Lee, Baoxin Li, Kewei Chen, Eric M. Reiman, Teresa Wu, Yi Su

**Affiliations:** ^1^ Arizona State University, Tempe, AZ USA; ^2^ ASU‐Mayo Center for Innovative Imaging, Tempe, AZ USA; ^3^ Banner Alzheimer’s Institute, Phoenix, AZ USA

## Abstract

**Background:**

Amyloid PET (Positron Emission Tomography) is crucial in detecting amyloid burden within the brain. However, the diversity of amyloid tracers and the scarcity of paired data significantly challenge the collaboration between cross‐center studies. In this research, we introduce a novel patch‐based 3D end‐to‐end image transformation model. This model works as a harmonization strategy, transferring the amyloid PET images from one tracer type to another.

**Method:**

51 florbetapir (FBP) and 604 PiB images from the Australian Imaging, Biomarkers and Lifestyle Study of Ageing (AIBL) were processed using established pipelines to extract regional standard uptake value ratios (SUVRs), mean cortical SUVRs (mcSUVRs), and SUVR images. 3D Cycle‐Consistent Generative Adversarial Networks (CycleGAN) was used to learn the end‐to‐end 3D image transformation using adversarial training strategies in conjunction with Resnet generators and multilayer discriminators within different tracer domains. Data augmentation techniques were applied to process the FBP images to balance the training samples and patch‐based learning was used throughout the experiment. The trained CycleGAN model was then applied to an independent dataset with 46 paired images from www.gaain.org/centiloid‐project for performance evaluation. Correlation analyses were conducted voxel‐wise and on mcSUVR, comparing the FBP/synthetic PiB to the true PiB data. The Structural Similarity Index Measure (SSIM) and Peak Signal‐to‐Noise Ratio (PSNR) were also evaluated between the synthetic and real PiB SUVR images.

**Result:**

The synthetic PiB SUVR images were visually more similar to real PiB SUVR images than FBP. Voxel‐wise correlation improved from 0.942 between FBP and real PiB to 0.958 between the virtual and real PiB SUVR image (p < 0.0001). The agreement of mcSUVR improved from r = 0.909 to r = 0.954 (p<0.001) in the independent test dataset. The SSIM and PSNR between synthetic and real PiB are 0.762 and 25.370 in the independent dataset.

**Conclusion:**

We proposed a novel end‐to‐end image transformation model for 3D PET image synthesis. The model finds the nonlinear mapping between different tracers and eliminates the requirement for paired training images. The result was confirmed using an independent dataset to demonstrate its effectiveness.